# Supplementary material for: Identification of common stria vascularis cellular alteration in sensorineural hearing loss based on ScRNA-seq
Source: BMC Genomics. 2024 Feb 27;25:213. doi: 10.1186/s12864-024-10122-7 (PMC10897997; doi:10.1186/s12864-024-10122-7)
Supplement: Supplementary file 2 — Supplementary Material 2. [file 12864_2024_10122_MOESM2_ESM.docx]

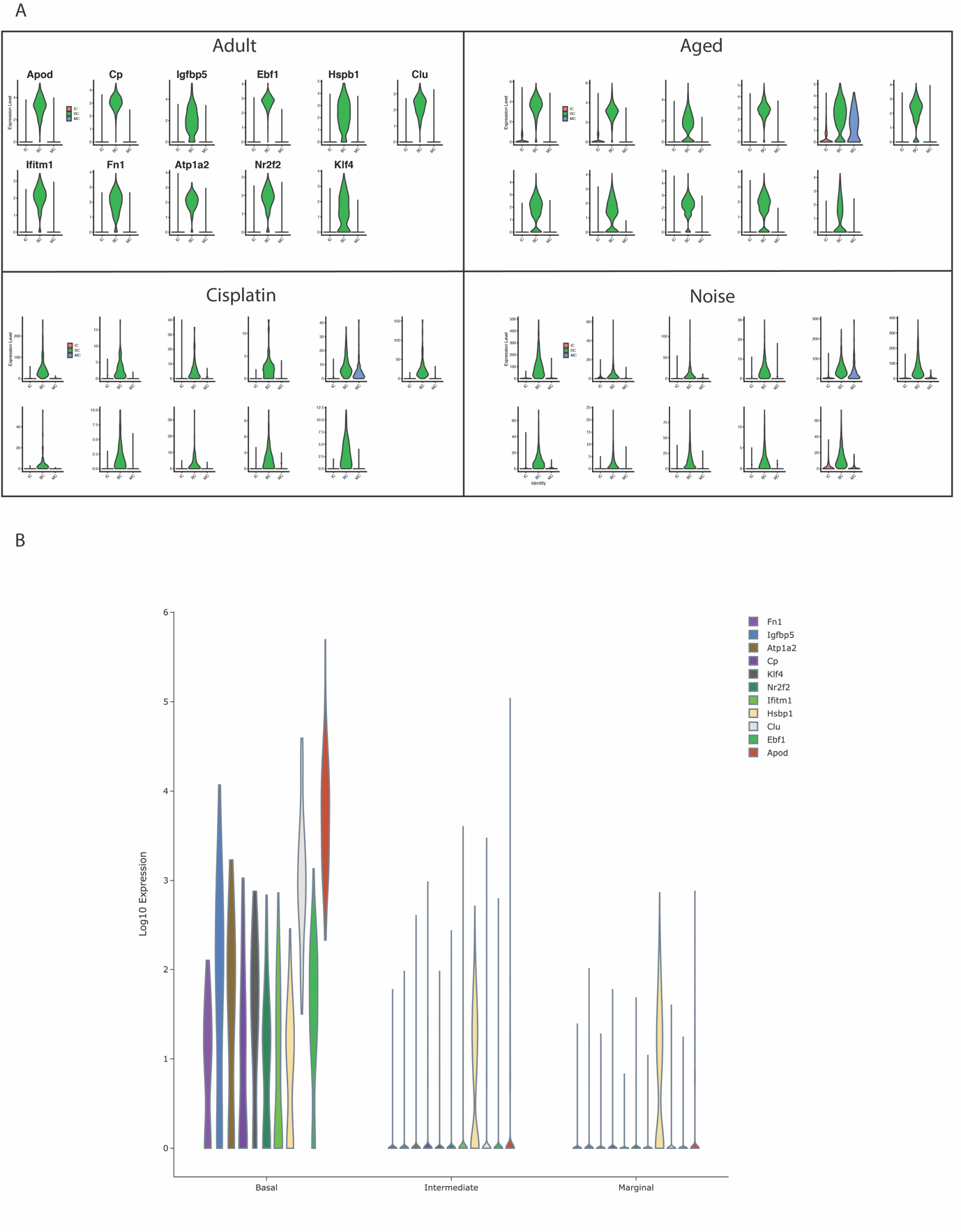


**Fig. S2 Common conserved genes expression within basal cell in different sensorineural hearing loss.**

The expression of BC-related genes in each original dataset: adult, age-related hearing loss (ARHL), noise-related hearing loss (NRHL), and cisplatin-related hearing loss (CRHL) **(A).** These genes are also supported in gEAR datasets **(B)**. BC, basal cell.
