# Supplementary material for: Identification of common stria vascularis cellular alteration in sensorineural hearing loss based on ScRNA-seq
Source: BMC Genomics. 2024 Feb 27;25:213. doi: 10.1186/s12864-024-10122-7 (PMC10897997; doi:10.1186/s12864-024-10122-7)
Supplement: Supplementary file 4 — Supplementary Material 4. [file 12864_2024_10122_MOESM4_ESM.docx]

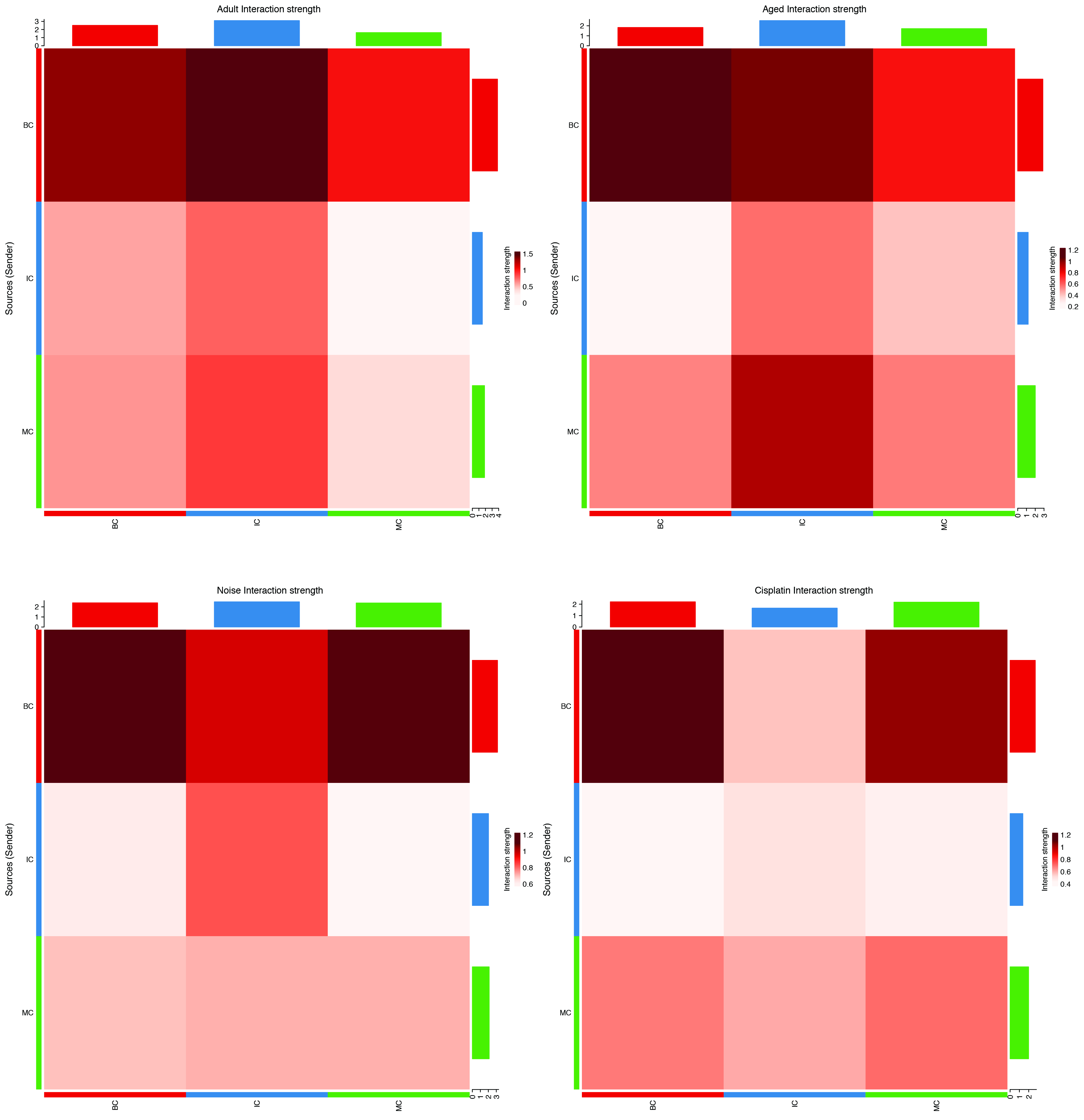


**Fig. S4 Heatmap of global alterations in signaling interactions between SV-specific cells under different treatments.** SV, stira vascularis.
