# Supplementary material for: Identification of common stria vascularis cellular alteration in sensorineural hearing loss based on ScRNA-seq
Source: BMC Genomics. 2024 Feb 27;25:213. doi: 10.1186/s12864-024-10122-7 (PMC10897997; doi:10.1186/s12864-024-10122-7)
Supplement: Supplementary file 6 — Supplementary Material 6. [file 12864_2024_10122_MOESM6_ESM.docx]

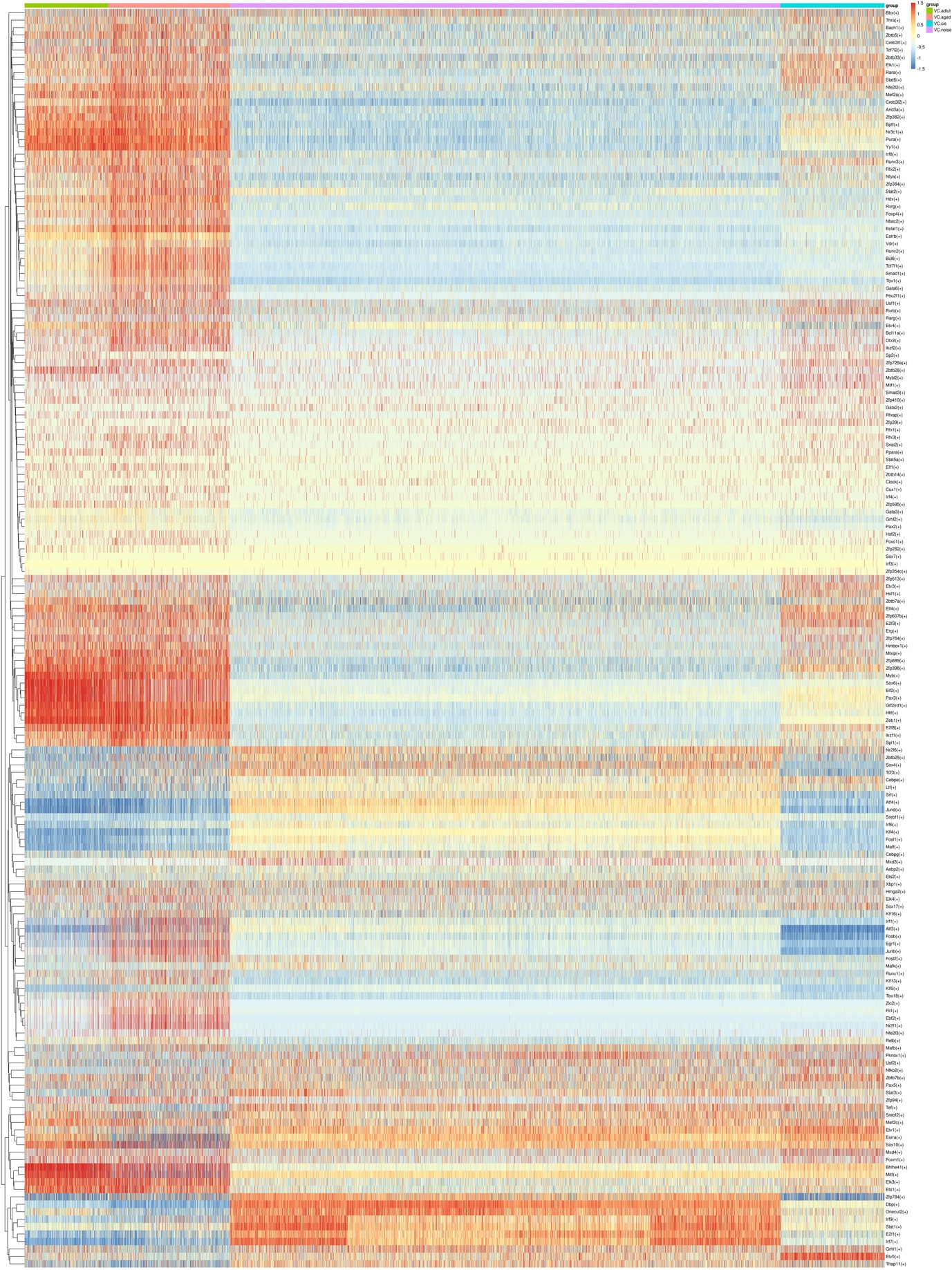


**Fig. S6 Heatmap of transcription factors of IC clustered in different sensorineural hearing loss.** IC, intermediate cell; SV, stira vascularis.
